# The positive effect of spermidine in older adults suffering from dementia after 1 year

**DOI:** 10.1007/s00508-023-02226-z

**Published:** 2023-06-07

**Authors:** Thomas Pekar, Aribert Wendzel, Reinhart Jarisch

**Affiliations:** 1 Biomedical Science, University of Applied Sciences Wiener Neustadt, Johannes-Gutenberg-Str. 3, 2700 Wiener Neustadt, Austria; 2 Gepflegt Wohnen Hart bei Graz GmbH, Pachern Hauptstr. 152d, 8075 Hart bei Graz, Austria; 3 FAZ Floridsdorfer Allergiezentrum, Pius-Parsch-Platz 1/3, 1210 Vienna, Austria

**Keywords:** Alzheimer, Nutrition, Biogen amines, Memory perfomance, CERAD-Plus

## Abstract

**Background:**

A positive effect of the effect of a 3-month oral spermidine intake on memory performance has already been demonstrated. The continuation of this study aimed to examine whether there could be observed an improvement in memory performance after one year.

**Method:**

45 residents of the nursing home “Gepflegt Wohnen” in Hart bei Graz, Styria, Austria, were given a daily dose of 3.3 mg spermidine in their diet for one year.

**Results:**

The comparison of the MMSE test results at baseline and after one year demonstrated a significant (p < 0.001) difference. The mean improvement is 5 points.

**Conclusion:**

The new results confirm the already proven positive effect of oral spermidine intake on memory performance.

## Introduction

Based on our promising results, according to which the oral intake of spermidine has already shown a positive effect on memory performance after 3 months [1], the nursing home group participating in the study decided to change the meals of all residents to a spermidine-rich diet. To ensure a daily intake of at least 3.3 mg spermidine per day, attention was paid to enriching as many meals as possible with spermidine. After 1 year, the residents of one house (Hart bei Graz) were again subjected to CERAD-Plus testing. We now present the results of this test.

## Material and methods

In total, 45 older individuals (MD 83, SD 9.5 years of age) participated in the follow up study. Participants were recruited via the director of nursing in the rest home in Hart bei Graz of the “Gepflegt Wohnen” group in Styria, Austria. People aged between 60 and 100 years were eligible for participation in the study. Furthermore, they had to not only take part in the CERAD-Plus test at the beginning and after 1 year but also continue their previous medication. Exclusion criteria comprised receiving antidementia medication, changing their previous medication, withdrawal by choice or participation in another study. Written informed consent was obtained from all participants in accordance with the Ethics Committee of the Medical University of Graz (30-280 ex 17/18). For those who were not able to understand the study, relatives or administrators provided consent.

Statistical analyses were carried out with the SPSS 26 statistical package (PASW, SPSS; IBM, Armonk, NY, USA). The Wilcoxon test for paired samples was applied for two-point comparison. The level of significance was set at a *p*-value of less than 0.05.

## Results

**Fig. 1:**
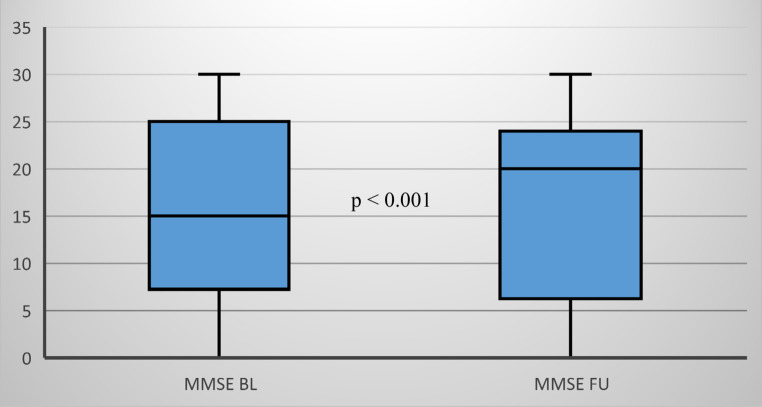
MMSE scores at the beginning (BL) and after 1 year (FU) daily intake of at least 3.3 mg spermidine, *n* = 45

The comparison of the results of the mini mental state examination (MMSE) showed a significant difference (*p* < 0.001). The median improvement was 5 points (Fig. 1).

Looking at the details, after 1 year an improvement in cognitive performance in 42% of the participants, a deterioration in 28% could be observed and 30% of the participants showed no change in MMSE.

A statistically significant improvement was also found in other categories of the CERAD-Plus test. In the “learn, recall and recognize a word list” section there was a medium improvement of 2 points (*p* = 0.047) and the evaluation of the item “sign and recall figures” also revealed an increase of 2 points (*p* = 0.039).

## Discussion

In a recently published study, in contrast to our results, no effect could be demonstrated after 1 year of spermidine intake [2]. We see one reason for this in the too low spermidine concentration of 0.9 mg, which is below the concentration we used in our control group in our most recently published work [1].

In a previous work we could show that there is a positive correlation between serum spermidine content and the MMSE score [3]. This fact was questioned in a letter to the editor due to too low R^2^ levels in the correlation analysis carried out [4]. A recently published study with a sample size of 3774 also described a correlation between serum spermidine levels and cognitive impairments [5]. In addition, the authors concluded that monitoring the spermidine levels may be helpful for reducing the incidence of cognitive impairments. We agree with this because, based on our findings, we believe that it is important to counteract low serum spermidine levels early on to reduce the risk of dementia.

In summary, the results after 1 year show a continuation of the positive effect of oral spermidine intake for patients with neurocognitive impairment.

## Limitations

As all subjects received the same dose of spermidine, blinding was not possible. The small number of participants should also be mentioned; however, as a small sample size usually results in a type II error and the null hypothesis was not accepted, this does not constitute a problem. Nevertheless, the results should be verified in a follow-up study with a larger sample.
